# Interactive effects of allergens and air pollution on respiratory health: A systematic review

**DOI:** 10.1016/j.scitotenv.2020.143924

**Published:** 2021-02-25

**Authors:** Holly C.Y. Lam, Deborah Jarvis, Elaine Fuertes

**Affiliations:** a National Heart and Lung Institute, Imperial College London, London, United Kingdom; b MRC Centre for Environment & Health, Imperial College, London, United Kingdom

**Keywords:** Interactions, Fungal Spore, Pollen, Pollution, Respiratory

## Abstract

**Background:**

Studies have demonstrated an adverse role of outdoor allergens on respiratory symptoms. It is unknown whether this effect is independent or synergistic of outdoor air pollutants.

**Methods:**

We systematically reviewed all epidemiological studies that examined interaction effects between counts of outdoor airborne allergens (pollen, fungal spores) and air pollutants, on any respiratory health outcome in children and adults. We searched the MEDLINE, EMBASE and Scopus databases. Each study was summarized qualitatively and assessed for quality and risk of bias (International Prospective Register for Systematic Reviews, registration number CRD42020162571).

**Results:**

Thirty-five studies were identified (15 timeseries, eight case-crossovers, 11 panels and one cohort study), of which 12 reported a significant statistical interaction between an allergen and air pollutant. Eight interactions were related to asthma outcomes, including one on lung function measures and wheeze, three to medical consultations for pollinosis and one to allergic symptoms (nasal, ocular or bronchial). There was no consensus as to which allergen or air pollutant is more likely to interact. No study investigated whether interactions are stronger in atopic individuals.

**Conclusion:**

Despite strong evidence from small experimental studies in humans, only a third of studies identified significant allergen-pollutant interactions using common epidemiological study designs. Exposure misclassification, failure to examine subgroups at risk, inadequate statistical power or absence of population-level effects are possible explanations.

## Highlights

• Whether allergen and pollutants interact to affect respiratory health is unknown.
• A systematic review of epidemiological studies was conducted.
• Studies in English were searched from the MEDLINE, EMBASE and Scopus databases.
• There was no consensus as to which allergen or pollutant is likely to interact.
• Evidence of allergen-pollutant interactions on respiratory health is weak.

## Graphical abstract

**Figure:**
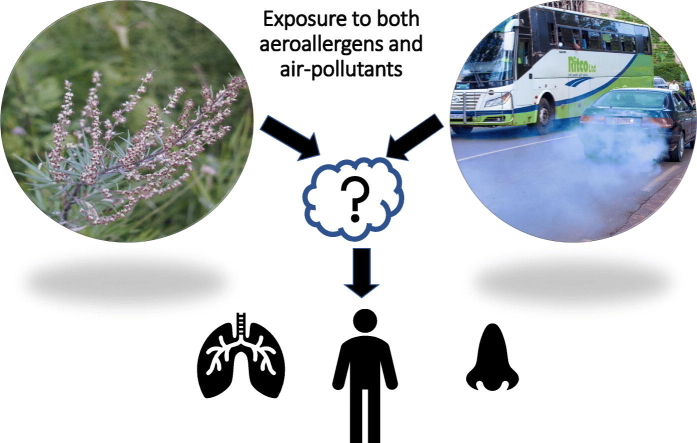

## 1 Introduction

The effects of outdoor airborne allergens on respiratory health have been evaluated in many epidemiological studies. A recent systematic review and meta-analysis concluded that outdoor pollen exposure is an important trigger for childhood asthma exacerbations requiring emergency department attendance (Erbas et al., 2018). Outdoor air pollutants, which coexist with airborne allergens, have also been widely studied for their adverse effects on respiratory health, in particular on asthma (Orellano et al., 2017). However, despite the substantial evidence supporting the existence of independent effects of allergens and air pollution on respiratory health, it remains unclear whether these environmental factors may also act synergistically.

Outdoor air pollutants have been shown to modify the allergenic potential of plant pollen and fungal spores via chemical modifications, increase allergen release from pollen grains and fungal spores and facilitate the transport of allergens into the airways. Air pollutants may also exert direct effects on individuals that could enhance the response to allergens, such as inducing epithelial damage which could increase oxidative stress and inflammation, and skewing the immune system towards an allergic response (Eguiluz-Gracia et al., 2020; Reinmuth-Selzle et al., 2017; Sedghy et al., 2018; Sénéchal et al., 2015; Smiljanic et al., 2019). Indeed, many small experimental studies conducted on humans have shown adverse synergistic effects of allergens and air pollutants on biological outcomes. For example, co-exposures to allergens and air pollution induce DNA methylation (Clifford et al., 2017), increase allergen-induced inflammatory and cellular immune responses (Hosseini et al., 2016), alter protein expression in lung (Mookherjee et al., 2018) and impair lung function (Wooding et al., 2019). Results from epidemiological studies, however, which assess allergen-pollutant interactions at ambient level appear less consistent (Anderson et al., 1998; Babin et al., 2008; Babin et al., 2007; Cakmak et al., 2012; Chen et al., 2016; Dales et al., 2004; Darrow et al., 2012; Erbas et al., 2012; Ghosh et al., 2012; Krmpotic et al., 2011; Lewis et al., 2000; Tham et al., 2017a; Tham et al., 2017b; Weisel et al., 2002).

Understanding allergen-pollutant interactions is especially relevant for urban areas where there are drives to increase green spaces and where the concentrations of some air pollutants tend to be high (Sousa-Silva et al., 2020). The assessment of co-exposures to pollen and pollutants in urban centres is an active area of research (Mücke et al., 2014; Ørby et al., 2015). As climate change is expected to increase pollen and fungal spore counts (Galán et al., 2016; Ziello et al., 2012) and influence their spread (Storkey et al., 2014), in addition to raising the concentrations of some air pollutants (e.g. carbon monoxide (CO), ozone, extreme dust events), it is vital that we understand how co-exposures to allergens and pollutants influence respiratory health (Schiavoni et al., 2017).

This systematic review expands on the work by Erbas et al. (2018) (focused specifically on asthma exacerbations and outdoor pollen) (Erbas et al., 2018), and is the first to review and summarize the current epidemiological evidence on the interaction effects of outdoor airborne allergens and air pollutants on any respiratory health outcome.

## 2 Material and methods

### 2.1 Search strategy

This systematic review was conducted following the Preferred Reporting Items for Systematic reviews and Meta-Analyses (PRISMA) (Moher et al., 2009, Moher et al., 2015) guidelines. A protocol was developed and uploaded to PROSPERO (International Prospective Register for Systematic Reviews, registration number CRD42020162571).

The literature was searched using three computerised bibliographic databases, MEDLINE, EMBASE and Scopus (search last updated April 27th, 2020). The search terms used in MEDLINE are listed in Table 1. Very similar but adapted terms were used in EMBASE (e.g. Emtree subheadings) and Scopus (Supporting Information, Tables S1–S2). The search terms were grouped into four categories (respiratory health, outdoor allergens, air pollution and study design) and linked with the Boolean operator “AND”. Reference lists of included studies were searched to find additional studies.

**Table 1:** Search terms used to identify articles in MEDLINE.

Category	Search terms
Respiratory health	MeSH terms: Asthma OR Bronchitis OR Cough OR Forced Expiratory Volume OR Pulmonary Ventilation OR Respiratory Sounds OR Rhinitis OR Respiratory Disorders OR Spirometry OR Vital Capacity Free text: airway* OR asthma* OR breath* OR bronchi* OR cough* OR exacerbation* OR hayfever OR hay fever OR lung OR pulmonary OR respiratory OR rhinoc* OR wheez*
Outdoor allergens	MeSH terms: Allergens OR Alternaria OR Cladosporium OR Fungi OR Pollen OR Spores, Fungal Free text: aeroallergen* OR aero allergen or aero allergens OR allergen* OR aspergillus OR cedar OR OR grass OR birch OR pollen* OR mould* OR mold* OR parietaria OR ragweed OR spore*
Air pollution	MeSH terms: Air Pollution OR Air Pollutants OR Ozone OR Nitrogen Dioxide OR Particulate Matter OR Sulfur Dioxide OR Traffic-Related Pollution OR Vehicle Emissions Free text: air quality OR carbon monoxide OR emission* OR exhaust OR freeway* OR highway* OR motorway* OR nitrogen dioxide OR particulat* OR particle* OR pollut* OR road* OR sulphur dioxide OR traffic* OR ozone
Study design	MeSH terms: Case-Control Studies OR Cross-Over Studies OR Cohort Studies OR Longitudinal Studies Free text: case control OR casecontrol OR case crossover OR cohort OR daily OR longitudinal OR panel OR timeseries OR time series

### 2.2 Study selection and eligibility criteria

All identified articles were downloaded into an electronic systematic review manager (Covidence, n.d.). Duplicates were deleted using the software's function. The titles and abstracts of the remaining articles were reviewed for eligibility by two researchers (HL, EF). Studies were retained if an outdoor allergen and air pollutant was mentioned in the abstract/title (or when it was suspected that both environmental factors were considered in the analysis). Subsequently, to identify studies that examined an interaction effect between an allergen and air pollutant, a text search was conducted for “interact*”, “stratif*” and “modi*”, and each publication's tables and figures were screened. The full texts of the publications retained were evaluated for data extraction and quality assessment by two researchers (HL, EF). Disagreements at all stages were resolved by a third author (DJ).

Studies had to meet the following three criteria to be included: (1) assess the interaction between at least one measured outdoor allergen and one air pollutant in relation to a respiratory health outcome in humans; (2) be conducted using a study design listed in Table 1; (3) be written in English. Studies conducted exclusively in laboratory settings or involving occupational allergens or indoor allergens were excluded. We included all potential interactions and made no assumptions of the nature of interactions.

Correspondingly, aligning with the PICO model, our population (P) of interest was all population groups, the intervention/exposure (I) was outdoor allergens and air pollutants, the comparison (C) made was different levels of exposure to these environmental factors, and the outcomes (O) considered were any respiratory health outcome.

### 2.3 Data extraction

We extracted the following information from each study: author names, publication year, study design, information on the study population (general/at risk, age, location, time period, sample size), allergens, air pollutants and health outcomes considered, outcome and exposure assessment methods, statistical analysis, adjustment factors, effect estimates and their corresponding 95% confidence intervals for the main effects of allergens and air pollutants, as well as their interaction, and whether effect modification by atopy was examined.

### 2.4 Study quality and risk of bias

The quality of each study was assessed using a modified version of a validated study quality instrument flexible enough to be applied to various study designs (Zaza et al., 2000). This instrument collects information on sample description and sampling method, outcome and exposure assessment, appropriateness of the statistical analysis and interpretation of the results. We further collected information on which allergen-pollutant interactions were tested (to assess potential reporting bias and multiple testing) and qualitatively, on the statistical power of the study to assess interactions. Consideration was given to study-level risk of bias and outcome-level risk of bias.

## 3 Results

### 3.1 Search results

**Fig. 1:**
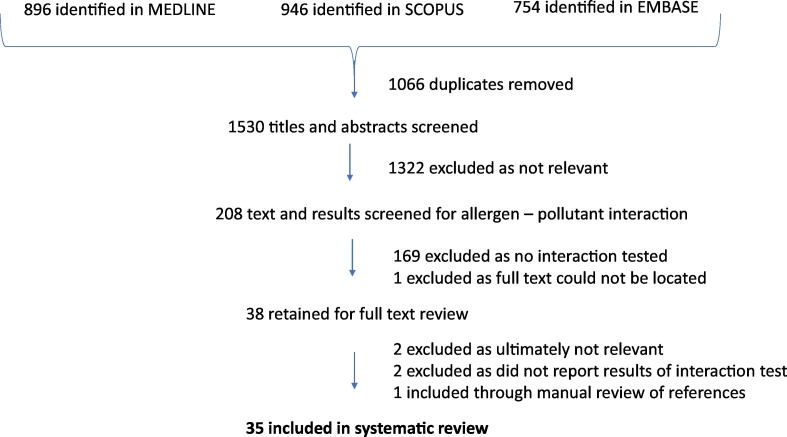
Flow chart of study selection.

The search yielded 1530 unique articles. Title and abstract screening led to the exclusion of 1322 articles. The remaining 208 articles were searched for whether an interaction effect between an allergen and air pollutant appeared to have been tested, which was the case in 38 articles. After fulltext reading, two articles were excluded as they ultimately did not test an interaction, two were excluded as they did not provide information on the results of the interactions tested and one new article was found through a manual search of reference lists and included, leading to a final number of 35 articles (flow chart provided in Fig. 1). The descriptive characteristics of these 35 studies and their findings related to allergen-pollutant interactions are summarized in Table 2 (additional information is provided in the Supporting Information, Table S3).

**Table 2:** Characteristics of studies ordered by study design (timeseries and case-crossover studies, panel studies, cohort study) and year.

Author, year	Type of study	Population, location	Time period	Outcome	Allergens	Air pollutants	Allergn - pollutant interactions	Atopic status
Guilbert et al., 2018	Timeseries	Residents of Brussels-Capital Region, Belgium	01/01/2008 to 31/12/2013	Daily number of asthma hospital admissions	Daily counts of 11 pollen taxa (alder, hazel, yew, Cupressaceae, ash, hornbeam, birch, oak, plantain, grasses, mugwort) and two fungal spore taxa (*Alternaria*, *Cladosporium*)	NO_2_, O_3_, PM_2.5_, PM_10_	Interactions tested between all allergens and air pollutants. Significant interactions found, notably risks higher for grass and birch pollen counts on days with high PM_10_ and O_3_ levels, respectively (p = 0.05). Interactions also found in the unexpected direction between *Alternaria* and PM_10_ and PM_2.5_, oak and NO_2_ and hazel and O_3_ (p = 0.06).	No information
Phosri et al., 2017	Case-crossover	Residents of Fukuoka City, Japan, attending 10 otolaryngology and ophthalmology clinics	2002 to 2012 (01/02–30/04 only)	Daily number of medical consultations for pollinosis	Daily counts of pollen (comprises Japanese cedar and cypress pollen)	PM_2.5_ mass and it's components (NO_3_^−^, non-sea-salt-SO_4_^2−^, sea-salt -Ca^2+^)	Interactions tested between pollen and all pollutants. Cumulative effect of pollen greater on days with high non-sea-salt Ca^2+^ (p < 0.01) and, for some models, non-sea-salt SO_4_^2^ and NO_3_^−^. Pollen effects (unexpectedly) greater on days with moderate (compared to high) total PM_2.5_ mass (p < 0.01).	No information
Sakata et al., 2017	Timeseries	Residents of Fukuoka City, Japan, attending four otorhinolaryngology clinics	1989 to 2012 for Clinic I and III, 1994–2012 for Clinic II, 1989–2001 for Clinic IV (February to April only)	Daily number of medical consultations for pollinosis	Daily counts of pollen (comprises Japanese cedar and cypress pollen)	Asian dust days (visibility <10 km), SPM	Interactions tested between Asian dust days and pollen and were consistently significant for all lags in 3 out of 4 clinics (p < 0.01). Interactions also tested between SPM and pollen and were significant for all four clinics during Asian dust-free days (p < 0.01).	No information
Tham et al., 2017a	Case-crossover	Asthmatic children (2–18 years) attending Campbelltown, Camden or Liverpool hospitals, Sydney, Australia	29/05/2008 to 03/05/2013	Daily number of asthma hospital admissions	Counts of total fungal spores and 20 fungal spore taxa	NO_2_, O_3_, PM_2.5_, PM_10_	Interactions tested between pollutants and allergens. None significant.	No information
Tham et al., 2017b	Case-crossover	Melbourne Air Pollen Children and Adolescent (2–17 years) study participants (hospitalized for asthma in Melbourne, Australia)	09/2009 to 12/2011	Daily number of asthma hospital admissions	Counts of total fungal spores and 12 fungal spore taxa (*Alternaria*, *Cladosporium*, *Ganoderma*, *Leptosphaeria*, *Pleospora*, *Sporormiella*, *Pithomyces*, smuts, *Coprinus*, *Drechslera*, *Stemphylium*, *Periconia*)	NO_2_, O_3_, PM_2.5_, PM_10_	Interactions tested between pollutants and allergens. None significant.	Information available for 630 participants. Analyses stratified by sensitization (8.9% to *Alternaria* only, 6.5% to *Cladosporium* only, and 13.7% to both).
Chen et al., 2016	Timeseries and case-crossover	Residents of Adelaide, South Australia	01/07/2003 to 30/06/2013	Daily number of asthma hospital admissions	Daily total pollen counts	NO_2_, O_3_, PM_2.5_, PM_10_,	Unclear which interactions tested but reported that none significant.	No information
Gleason et al., 2014	Case-crossover	Children (3–17 years) in New Jersey, USA	2004 to 2007 warm season (01/04 to 30/09 only)	Daily number of asthma emergency department visits	Daily counts of tree pollen, weed pollen, grass pollen and ragweed	O_3_, PM_2.5_	Stated that interactions tested between quintiles of O_3_ and PM_2.5_ with high vs low categories of each pollen type, although no interaction terms formally reported. Stratified models indicate effect of O_3_ and PM_2.5_ greater on days with high 3-day average weed pollen counts.	No information
Konishi et al., 2014	Case-crossover	Residents of Chiyoda ward, Tokyo, Japan, attending one general practice	2001 to 2011 (January to May only)	Daily number of initial doctor consultations for pollinosis	Daily counts of pollen (comprises Japanese cedar and cypress pollen)	CO, NO_2_, O_3,_ PM_2.5_, SO_2_, SPM	Interactions tested between pollen counts and PM_2.5_ and SPM. Effect of 5-day average cumulative pollen count greater on days with high PM_2.5_ (consistently) or SPM levels (less consistently), compared to days with moderate pollutant levels. Results less clear when considering single-day pollen effects.	No information
Cakmak et al., 2012	Timeseries	Residents of 11 cities in Canada.	01/04/1994 to 31/03/2007	Daily number of asthma hospital admissions	Daily counts of weed, trees and grass pollen and Basidiomycetes, Ascomycetes and Deuteromycetes fungal spores.	CO, NO_2_, O_3,_ PM_2.5_, PM_10_, SO_2_	Interactions tested between all pollutants and all allergens. Effect of allergens greater on days with high air pollution levels in several cases, especially for PM_10_. Interaction terms significant (p < 0.05) between ascomycetes and CO and PM_10_, basidiomycetes and CO, NO_2_, SO_2_ and PM_10_, deuteromycetes and CO, NO_2_, SO_2_ and PM_10_, trees and PM_2.5_, weeds and PM_10_	No information
Darrow et al., 2012	Case-crossover	Residents of Atlanta metropolitan area, USA, attending 41 acute-care hospitals	1993 to 2004 Analyses limited to pollen season for each taxa (2–5 months).	Daily number of emergency department visits for asthma and wheeze	Daily counts of four tree pollen taxa (Betulaceae, Cupressacea, Quercus, Pinaceae), grasses and Ambrosia	CO, NO_2_, O_3_, PM_2.5_, PM_10_, SO_2_	Interactions between O_3_ and Quercus and grass pollen count tested, but neither significant.	No information
Erbas et al., 2012	Timeseries	Children (<15 years) living in Melbourne, Australia	01/09/2003 to 31/12/2003	Daily number of asthma emergency department visits for asthma	Daily counts of grass pollen	NO_2_, O_3_, airborne particle index	Interactions tested between grass pollen count and the Airborne Particle index and NO_2_. Neither significant.	No information
Ghosh et al., 2012	Timeseries	Residents of Kolkata, India, with a diagnosed respiratory allergy attending a hospital allergy clinic (catchment area of 2 hospitals included)	2004 to 2009	Number of asthma-related hospital admissions recorded in 10-day time-slots.	Daily counts of five pollen types (*Areca*, *Carica*, *Cheno-Amnthaceae*, *Cocos*, *Phoenix*, *Cyperaceae*, *Poaceae*), 7 fungal types (*Alternaria*, *Aspergilli*, *Basidiospores*, *Cladosporium*) and total fungal spores	NO_2_, SO_2_, SPM, respirable particulate matter, O_3_ (latter only for two years)	Unclear which interactions tested but reported that none significant.	No information
Krmpotic et al., 2011	Timeseries	Residents of Zagreb, Croatia	01/01/2004 to 31/12/2006	Daily number of asthma hospital admissions	Daily alder, hazel, birch, hornbeam, oak, grasses and ragweed pollen counts	CO, NO_2_, PM_10_	Interactions tested between all pollutants and pollen variables. None significant.	No information
Babin et al., 2008	Timeseries	Residents of Washington, DC, USA, on Medicaid health plan	01/10/1994 to 22/11/2005	Daily number of asthma-related acute care visits	Daily tree, grass, weed pollen counts and total mold spore counts.	O_3_, PM_2.5_, PM_10_	Unclear which interactions tested but reported that none significant.	No information
Babin et al., 2007	Timeseries	Residents of Washington, DC, USA.	10/2001 to 09/2004 (except 12/2002)	Daily number of asthma-related pediatric emergency department visits and admissions	Daily tree, grass, weed pollen counts and total mold spore counts	O_3_, PM_2.5_	Interactions tested between both pollutants and all allergens but none significant.	No information
Carracedo-Martinez et al., 2008	Case-crossover	Residents of the City of Vigo, Spain	1996–1999	Daily number of medical emergency calls for cardiovascular and respiratory causes	20 types of pollen counts	Black smoke, SO_2_	Unclear which interactions tested but reported that none significant.	No information
Villeneuve et al., 2006	Timeseries	Elderly (≥65 years) residents of Toronto, Canada.	01/01/1995 to 31/12/2000	Daily number of primary care visits for allergic rhinitis	Daily ragweed counts	NO_2_, O_3_, PM_2.5_, PM_10_, PM_2.5__–__10_, SO_2_	Interactions tested between ragweed and all pollutants except O_3_. None significant.	No information
Dales et al., 2004	Timeseries	Residents of 10 cities in Canada.	01/04/1993 to 31/03/2000 (only months with pollen/fungal counts >0)	Daily number of asthma hospital admissions	Daily counts of weed, trees and grass pollen and Basidiomycetes, Ascomycetes and Deuteromycetes fungal spores.	NO_2_, O_3_, SO_2_, coefficient of haze	In combined analyses, only interaction term between O_3_ and tree pollen count was significant (p < 0.05). City-specific interactions also observed between O_3_ and certain allergens (basidiomycetes in 3 cities, deuteromycetes in 3 cities, weeds in 3 cities, trees in 4 cities, grass in 1 city).	No information
Lierl and Hornung, 2003	Timeseries	Residents of Cincinnati, USA	04/1996 to 10/1996 and 04/1997 to 10/1997 (08/1996 excluded)	Daily number of asthma hospital visits (includes visits and admissions combined)	Daily counts of total pollen and fungal spores	O_3_, PM_10_	Interactions tested between pollen counts and air pollutants. No interaction terms formally reported. Stratified models indicate effect of pollen counts greater on high (> 33 μg/m^3^) PM_10_ days.	No information
Sunyer et al., 2002	Case-crossover	Residents of Barcelona, Spain, who attended emergency department for asthma between 1985 and 1989 in the four largest hospitals (covers 80% of population)	1985 to 1995	Number of overall and cause-specific mortality (including respriatory mortality)	Weekly counts of total pollen, grass pollen, total fungal spores, *Cladosporium*, *Alternaria*, *Epicoccum* and *Helminthosporium*	Black smoke, CO, NO_2_, O_3_, SO_2_	Interactions tested between pollutants and allergens. None significant.	No information
Weisel et al., 2002	Timeseries	Residents of central and northern New Jersey, USA	01/05/1995 to 31/08/1995	Daily number of asthma hospital visits and admissions	Daily counts of total pollen and total fungal spores	O_3_	Unclear which interactions tested but reported that none significant	No information
Lewis et al., 2000	Timeseries	Residents of Derby, England	01/1993 to 12/1996	Daily number of asthma hospital admissions and visits (assessed separately)	Daily counts of grass and birch pollen, hyaline basidiospores, coloured basidsiospores, *Didymella*, *Alternaria*, and *Cladosporium*	Black smoke, O_3_, NO_2_	Interactions tested between all pollutants and pollen variables. None significant.	No information
Anderson et al., 1998	Timeseries	Residents of London, UK	1987 to 1992	Daily number of asthma hospital admissions	Daily birch, grass and oak pollen counts	Black smoke, NO_2_, O_3_, SO_2_,	Interactions tested between all three allergens and pollutants significant in unipollutant models (O_3_ and SO_2_), restricted to warm season. Significant interaction found between SO_2_ and grass pollen counts in 0–14 age group in warm season (p < 0.01). Interactions also found between ozone and birch and ozone and oak in unexpected direction in all-ages analysis in the warm season (p < 0.001)	No information
DellaValle et al., 2012	Panel	Children (4–12 years) with asthma living in Connecticut, Massachusetts and New York, USA	Recruited 2000 to 2003, each followed for one year.	Daily record of asthma symptoms of wheeze, night symptoms, shortness of breath, chest tightness, persistent cough and rescue medication use^a^	Daily counts of total pollen, and tree, grass and weed pollen	NO_2_, O_3_, PM_2.5_, SO_2_	Interactions tested between O_3_ and all pollen variables, but none significant.	Information available for 319 (74%) subjects. Analyses stratified by sensitization to grass (26%) and weed (22%) pollen.
Chen et al., 2011	Panel	Children (mean age 10.6 years) of three elementary and two middle schools in Taipei Country, Taiwan	09/2007 to 06/2008	Monthly FVC, FEV_1_, FEF_25_, FEF_50_, FEF_75_, FEF_2575_	Daily total fungal spore counts in week of lung function measurement	CO, NO_2_, O_3_, PM_2.5_, PM_coarse_, SO_2_	Unclear which interactions tested but reported that none significant.	No information
Jalaludin et al., 2004	Panel	Primary school children (mean age 9.6 years) with a history of wheeze attending six primary schools in western and southwestern Sydney.	01/02/1994 to 31/12/1994	Daily record of respiratory symptoms (wheeze, wet cough, dry cough), asthma medication use, visit to a doctor for asthma^a^	Daily counts of total pollen and *Alternaria*	NO_2_, O_3_, PM_10_	Interaction terms tested between air pollutants and counts of total pollen and Alternaria, but none significant.	No information
Delfino et al., 2002	Panel	Children/adolescents (9–19 years) with asthma living in Alpine California, USA	01/03/1996 to 30/04/1996	Daily record of asthma symptoms that interfere with daily activities^a^	Daily counts of total pollen and fungal spores	NO_2_, O_3_, PM_10_	Interactions tested between pollutants and allergens but none significant.	77.3% sensitized to pollens or fungi.
Just et al., 2002	Panel	Children (mean age 10.9 years) diagnosed with asthma who are outpatients at a hospital in Paris, France	01/04/1996 to 30/06/1996	Daily record of incident and prevalent asthma attacks, nocturnal cough, wheeze, irritation symptoms, respiratory infections, use of β2-agonist and morning and daily variability in PEFR^a^	Daily counts of total pollen	Black smoke, NO_2_, O_3_, PM_13_ (SO_2_ available but levels too low for analysis)	Interaction terms tested between O_3_ and pollen counts. Interaction term significant for incident asthma attacks (p = 0.002).	90.2% were atopic.
Ross et al., 2002	Panel	Asthmatics living in East Moline, USA	24/05/1994 to 25/10/1994	Daily record of morning and evening PEFR, symptom scores frequency of asthma attacks and asthma medication use^a^	Daily counts of total, grass and ragweed pollen, as well as counts of total, *Alternaria*, *Cladosporium*, *Curvularia*, *Drechslera* and *Epicoccum* spores.	O_3_, PM_10_ (only every 6th day), SO_2_	Interactions tested between O_3_ and allergens. None significant.	41–46% sensitized to trees, grasses or ragweed. Analyses stratified by sensitization.
Higgins et al., 2000	Panel	Asthmatics or those with chronic obstructive pulmonary disease who react to methacholine attending two general practices in Halton Health District, UK	28 days from August to mid-September 1991	Daily mean and variability in PEFR, presence of wheeze symptoms (data for dyspnoea, cough, throat irritation, eye irritation not usable for analyses)^a^	Daily counts of total fungal spores (measured pollen counts too low for analyses)	NO_2_, O_3_	Interactions tested between total fungal spore counts and O_3_ and NO_2_. Interactions observed between fungal spores and O_3_ for daily mean and variability in PEFR and wheeze symptoms (p < 0.02). Interaction also found between fungal spores and NO_2_ for wheeze but in unexpected direction (p < 0.05).	63% atopic. Analyses stratified by sensitization.
Jalaludin et al., 2000	Panel	Primary school children (mean age 9.6 years) with a history of wheeze attending six primary schools in western and southwestern Sydney	01/02/1994 to 31/12/1994	Daily record of evening PEFR (daily mean deviation of 3 measurements and maximum)	Daily counts of total pollen and *Alternaria*	NO_2_, O_3_, PM_10_	Unclear which interactions tested but reported that none significant	No information
Delfino et al., 1997	Panel	Asthmatics living in Alpine California, USA	09/05/1994 to 03/07/1994	Daily record of responses for asthma symptom severity and inhaler use and morning and evening PEFR^a^	Daily counts of total pollen and fungal spores, as well as 12 fungi types (*Alternaria*, *Cladosporium*, *Helminthosporium*, *Aspergillus* and *Penicillium*, *Coprinus*, *Periconia*, *Botrytis*, total ascospores, total basidiospores, hyphal fragments, rusts, unidentified spores).	O_3_, PM_10_	Interactions tested between pollen counts, PM_10_ and O_3_ with fungal spore counts for all outcomes, but none significant.	All subjects sensitized to ≥1 pollen extract. Analyses conducted among 16 subjects sensitized to ≥1 of five skin prick tests for fungal spore taxa.
Delfino et al., 1996	Panel	Children/adolescents (9–16 years) with asthma living in Mesa region in San Diego, USA	20/09/1993 to 31/10/1993	Daily record of responses for asthma symptom severity and inhaler use^a^	Daily counts of total pollen and fungal spores, as well as 5 fungi types (*Alternaria*, *Cladosporium*, *Helminthosporium interseminatum*, *Aspergillus*, *Penicillium*).	HNO_1_, HNO_3_, O_3_, PM_2.5_, sulfate and nitrate fractions of PM_2.5_	Interactions tested between O_3_ and the fungal count variables, but none significant.	All subjects sensitized to ≥1 pollen extract. Analyses conducted among 10 subjects sensitized to ≥1 of five skin prick tests for fungal spore taxa.
Jones et al., 1994	Panel	Children (mean age 10.6 years) with a respiratory disorder (95% had asthma) attending two private practice offices and one pediatric clinic in Baton Rouge, Louisiana	14/06/1990 to 30/09/1990	Daily record of PEFR, respiratory symptom count and rating, activity time and rating, asthma/COPD medication count^a^	Daily counts of total pollen and fungal spores	NO_2_, O_3_	Two-way interactions tested between both allergens and pollutants, as well as one-three way interaction between O_3_, NO_2_ and fungal spore counts. None significant.	No information
Kanatani et al., 2016	Longitudinal cohort	Pregnant women (taking part in a larger cohort study) living in Kyoto, Toyama and Tottori, Japan	10/2011 to 11/2011, 02/2012 to 05/2012, 10/2012 to 11/2012, 02/2013 to 05/2013	Any allergic symptom (nasal, ocular or bronchial; symptom score > 0)^a^	Daily counts of pollen (comprises Japanese cedar and cypress pollen)	Asian dust days (dust level ≥ 0.07/km) compared to randomly selected control days (dust level < 0.07/km)	Interaction term tested between dust days (yes/no) and pollen counts, and was significant (p < 0.001)	57.2% sensitized to Japanese cedar pollen. Analyses stratified by sensitization.

CO = carbon monoxide; NO_2_ = nitrogen dioxide; O_3_ = ozone; PEFR: peak expiratory flow rate; PM_2.5_ = particulate matter ≤2.5 μm in aerodynamic diameter; PM_10_ = particulate matter ≤10 μm in aerodynamic diameter; SO_2_ = sulphur dioxide; SPM = suspended particulate matter.

a Self-reported.

### 3.2 Study characteristics

Of the 35 included studies, there were 15 timeseries, eight case-crossovers, 11 panel and one cohort study. The most common health outcome was daily number of asthma hospital emergency department admissions or visits, assessed in timeseries and case-crossover studies (n = 17). Daily records of self-reported asthmatic, allergic (nasal, ocular or bronchial) or respiratory symptoms (n = 10) followed by changes in daily measurements of lung function (n = 7) were the next most frequently investigated outcomes, assessed in panel studies. All timeseries and case-crossover studies used administrative respiratory health data whereas information on respiratory symptoms was collected using self-completed (or parent-completed) questionnaires or diaries in the panel studies. Most studies were conducted in the United States (n = 12), Australia (n = 6), Japan (n = 4), Canada (n = 3) and the United Kingdom (n = 3). Fourteen studies were conducted in children/adolescents, four exclusively in adults and 17 in both children and adults.

Interactions with total pollen and total fungal spore counts were examined in 12 and 13 studies, respectively, whereas specific pollens (e.g. grasses, ragweed) and fungal types were examined in 22 and 13 studies, respectively. Most studies (n = 24) defined daily allergen exposure for the entire study area as the daily mean count obtained from one monitoring site. Measurements were most commonly made using a volumetric trap (Burkard/Hirst), although the measurement device was not always reported. A few studies used data from several monitoring sites to calculate daily exposures for the study area (two (Jalaludin et al., 2000, Jalaludin et al., 2004), three (Ross et al., 2002) or nine (Konishi et al., 2014) monitoring sites) or assigned daily exposures to home addresses using data from the closest monitoring site from participants' home (Kanatani et al., 2016) or a spatial-temporal model (DellaValle et al., 2012). Only the data provider of the allergen information was provided in five studies (Babin et al., 2008; Babin et al., 2007; Just et al., 2002; Krmpotic et al., 2011; Weisel et al., 2002).

Similar methods were used to assign air pollution exposures as those used to assign allergen exposures, although usually data from a larger number of monitoring sites were available. Notable exceptions included the use of a spatial-temporal model which provided daily estimates of pollutants at a 12 km square grid scale (Gleason et al., 2014) and personal samplers to assign ozone exposures in two studies (Delfino et al., 1996, Delfino et al., 1997).

### 3.3 Allergen and air pollutant interactions

Of the 35 studies that examined allergen-pollutant interactions (summarized in Tables 2 and S3), 12 reported at least one significant interaction effect: nine in timeseries and case-crossover studies, two in panel studies and one in a cohort study.

#### 3.3.1 Timeseries and case-crossover studies

Six timeseries or case-crossover studies reported allergen-pollutant interactions for daily number of asthma hospital admissions or visits.

In a five-year timeseries study in London, two pollutants (ozone and SO_2_) showed main effects on daily number of asthma hospital admissions, and interactions were tested with daily birch, grass and oak pollen counts. Only the interaction between SO_2_ and grass pollen counts was statistically significant (and in the hypothesized direction) among 0–14 year-olds in the warm season (April to September) (p < 0.01). Two other interactions were noted in the all-ages analysis in the warm season (between ozone and birch and ozone and oak, p < 0.001), but these were in the unexpected direction, as results suggested stronger adverse effects of ozone on days with no pollen (Anderson et al., 1998).

In a second timeseries study using data on daily weed, tree and grass pollen counts, counts of three fungal classes (Basidiomycetes, Ascomycetes, Deuteromycetes), as well as NO_2_, ozone, SO_2_ concentrations and a coefficient of haze in ten Canadian cities over seven years, an interaction term between ozone and tree pollen counts was significant for overall daily number of asthma hospital admissions in analyses including all data (p < 0.05). Additional interactions were observed between ozone and counts of certain allergens in city-specific analyses (basidiomycetes in three cities, deuteromycetes in three cities, weeds in three cities, trees in four cities, grass in one city) (Dales et al., 2004). A further Canadian timeseries study which examined the same allergens reported a larger effect of several fungal and pollen allergen counts on days with high air pollution concentrations, especially PM_10_. Interaction terms were significant (p < 0.05) between ascomycetes counts and CO and PM_10_, basidiomycetes counts and CO, NO_2_, PM_10_, and SO_2_, deuteromycetes counts and CO, NO_2_, PM_10_, and SO_2_, tree pollen counts and PM_2.5_, as well as weed pollen counts and PM_10_ (Cakmak et al., 2012). It is worth noting the substantial overlap in data between these two Canadian studies: Dales et al. (2004) (Dales et al., 2004) reported allergen-pollutant interactions over seven years (01/04/1993 to 31/03/2000) in ten cities and Cakmak et al. (2012) (Cakmak et al., 2012) examined the same allergens over 13 years (01/04/1994–31/03/2007) in the same ten cities plus one other.

In a six-year timeseries study, interactions were tested between daily counts of 11 pollen taxa and two fungal spore taxa and NO_2_, ozone, PM_2.5_, PM_10_ in relation to overall daily number of asthma hospital admissions in the Brussels-Capital Region, Belgium (Guilbert et al., 2018). Several significant interactions were found, although only two were in the expected direction; the adverse effects of grass and birch pollen counts were higher on days with high PM_10_ and ozone concentrations, respectively (p = 0.05). Results in the opposite direction (i.e. lower asthma hospitalizations associated with increasing allergen counts) were observed for Alternaria on days with high PM_10_ and PM_2.5_ concentrations, oak on days with high NO_2_ concentrations and hazel on days with high ozone concentrations (p = 0.06).

Although no formal interaction terms were provided, another timeseries study reported that the adverse effect of total pollen counts on daily number of asthma hospital visits in children (including 14 months of combined data on visits and admissions from Cincinnati, USA) was greater on high PM_10_ days (but not high ozone days) using stratified analyses (Lierl and Hornung, 2003). Of note, the authors state “We found significant interactions between season and pollen count and between pollen count and measured particulate level” but do not present the results of the interaction term. A three-year case-crossover study also used stratified analyses to demonstrate increasing adverse effects of ozone and PM_2.5_ on daily number of asthma emergency department visits in children on days with high (but not low) three-day average weed counts during the warm season in New Jersey, United States. No diffference in ozone and PM_2.5_ effects were observed on high tree, grass or ragweed pollen days (Gleason et al., 2014). Although no formal interaction tests were reported for these latter two studies (Gleason et al., 2014; Lierl and Hornung, 2003), we here consider them as having found allergen-pollutant interactions based on the author's interpretation of the data.

In constrast to the above six studies which reported significant allergen-pollutant interactions on daily number of asthma hospital admissions or visits, eleven similar timeseries or case-crossover studies did not find significant interactions (Chen et al., 2016; Darrow et al., 2012; Erbas et al., 2012; Ghosh et al., 2012; Lewis et al., 2000; Tham et al., 2017a; Tham et al., 2017b; Babin et al., 2008; Babin et al., 2007; Krmpotic et al., 2011; Weisel et al., 2002). Combined, these studies cover various parts of the world and assessed interactions between both total and specific pollen and fungal spore counts and several pollutants. Although a few had rather short time-frames (≤3 years; (Babin et al., 2007; Erbas et al., 2012; Krmpotic et al., 2011; Tham et al., 2017b; Weisel et al., 2002)), this was not a universal trend in this group of studies reporting null findings for allergen-pollutant interactions.

Three Japanese studies examined interactions between measures of air pollutants and pollen counts comprising Japanese cedar and Japanese cypress pollen on medical consultations for pollinosis, all of which reported significant interactions. First, in a case-crossover study using ten years of data from one ward in Tokyo, the adverse effect of 5-day average cumulative pollen was greater on days with high PM_2.5_ mass compared to days with more moderate levels (p = 0.02) (Konishi et al., 2014). Results were similar but less consistent for suspended particulate matter. Second, in a timeseries analysis, consistent interactions between pollen counts and Asian dust days (visibility <10 km) were reported in three out of four clinics in Fukuoka City (p < 0.01), as well as between pollen counts and suspended particulate matter in all four clinics during Asian dust-free days (p < 0.01) (Sakata et al., 2017). Third, interactions were tested between pollen counts and PM_2.5_ total mass and it's components (NO_3_^−^, non-sea-salt-SO_4_^2−^, sea-salt -Ca^2+^) in a ten-year timeseries study using data from ten clinics in Fukuoka City (Phosri et al., 2017). The adverse two-day cumulative effect of pollen was greater on days with high non-sea-salt Ca^2+^ levels (p < 0.01) and less consistently, high non-sea-salt SO_4_^2^ and NO_3_^−^ levels. However, the adverse effect of pollen was also unexpectedly greater on days with moderate compared to high total PM_2.5_ mass (p < 0.01).

Finally, one four-year timeseries study in Vigo, Spain, assessed interactions with various pollen counts and black smoke and SO_2_ on daily number of medical emergency calls for cardiovascular and respiratory cases (Carracedo-Martinez et al., 2008), one six-year timeseries study of elderly residents in Toronto, Canada, assessed interactions with ragweed counts and several air pollutants on daily number of primary care visits for allergic rhinitis (Villeneuve et al., 2006), and one ten-year case-crossover study of asthmatics in Barcelona, Spain, assessed interactions with pollen and fungal spore counts and several pollutants on overall and case-specific mortality (Sunyer et al., 2002). All three of these analyses yielded null findings.

#### 3.3.2 Panel and cohort studies

The six timeseries and case-crossover studies that reported allergen-pollutant interactions on daily number of asthma hospital visits or admissions are weakly supported by the results of only two panel studies. First, among 35 adult patients with asthma or chronic obstructive pulmonary disease followed for four weeks during late summer (August to mid-September) in the United Kingdom, increasing total fungal spore counts were associated with reduced daily mean peak expiratory flow rate and increased daily variability in peak expiratory flow rate and reported wheeze symptoms, and these effects were greater the higher the prior (24–48 h) ozone level (p < 0.02) (Higgins et al., 2000). However, an interaction effect between fungal spore counts and NO_2_ for wheeze was also found in the unexpected direction (p < 0.05). Second, in a three-month panel of 82 children diagnosed with asthma (90.2% atopic and all outpatients at a hosptial in Paris, France), an interaction was found between ozone and total pollen count for daily records of self-reported incident asthma attacks (p = 0.002). Significant interactions were not found for daily records of self-reported respiratory symptoms, medication use or measures of peak expiratory flow rates (Just et al., 2002).

Of the nine panel studies that did not find significant allergen-pollutant interactions, seven assessed asthma or respiratory symptoms or medication use as outcomes (five included children/adolescents (Delfino et al., 2002; Delfino et al., 1996; DellaValle et al., 2012; Jalaludin et al., 2004; Jones et al., 1994) and two included participants of all ages (Delfino et al., 1997; Ross et al., 2002)), and five considered peak expiratory flow rate measures as outcomes (three included asthmatics of all ages (Delfino et al., 1997; Jones et al., 1994; Ross et al., 2002), one included children with wheeze (Jalaludin et al., 2000) and one included children with asthma, allergic rhinitis or healthy controls (Chen et al., 2011)).

Finally, in a Japanese cohort study of 3328 pregnant women followed for a total of 12 months of Asian dust seasons (February to May and October to November) (over three years), an interaction (p < 0.001) was found between the presence of Asian dust days (dust level ≥ 0.07/km) and pollen counts for developing any allergic symptom (nasal, ocular or bronchial symptoms, symptom score > 0) reported using a mobile phone questionnaire (Kanatani et al., 2016).

### 3.4 Effect modification by atopy

Of the total 35 studies, nine presented information on the atopic status of participants (six panel, one case-crossover and one cohort study) Of these nine, one included nearly exclusively atopic individuals (90.2%, (Just et al., 2002)) and eight included a mix of atopic and non-atopic individuals (Delfino et al., 2002; Delfino et al., 1996, Delfino et al., 1997; DellaValle et al., 2012; Higgins et al., 2000; Kanatani et al., 2016; Ross et al., 2002; Tham et al., 2017b). Of these latter eight studies which could conduct analyses stratified by atopic status, four reported some stronger main effects of allergens or air pollutants among atopic subgroups (Delfino et al., 1997; DellaValle et al., 2012; Kanatani et al., 2016; Tham et al., 2017b) but none assessed whether allergen-pollutant interactions may be stronger among atopic individuals.

### 3.5 Quality assessment and risk of bias

Most studies were of good quality (quality assessment conducted summarized in the Supporting Information, Table S4). Selection bias is unlikely in the timeseries and case-crossover studies as these tended to include hospitals comprehensively covering the intended study areas. Loss to follow-up is more of a concern for the panel studies, although only two included <80% of initially recruited participants (Delfino et al., 1996; Ross et al., 2002). Variation in effect estimates by individual-level factors was rarely considered. Only one study assessed allergen-pollutant interactions by age group (Anderson et al., 1998) and none examined allergen-pollutant interactions by atopic status. Multiple testing and reporting bias are likely as multiple lags and several allergen-pollutant combinations were frequently tested but interaction effect estimates or p-values reported for only a subset of these analyses. Although difficult to assess, limited statistical power to test allergen-pollutant interactions is suspected in several studies.

## 4 Discussion

In this systematic review including 35 epidemiological studies that tested allergen-pollutant interactions in the context of respiratory health, we identified 12 studies that found a significant statistical interaction. Eight interactions were related to asthma outcomes, including one on lung function measures and wheeze, three to medical consultations for pollinosis and one to allergic symptoms. There was no consistency as to which allergen or air pollutant is more likely to interact and no study investigated whether allergen-pollutant interactions may be stronger in atopic individuals. There were no apparent systematic differences in terms of population studied (e.g. adults vs. children), outcome and exposures examined, assessment strategies used, sample size or length of follow-up between the 12 studies that reported finding an allergen-pollutant interaction and the 23 that did not.

As the relative majority (six) of allergen-pollutant interactions were identified for daily number of asthma hospital admissions or visits (Anderson et al., 1998; Cakmak et al., 2012; Dales et al., 2004; Gleason et al., 2014; Guilbert et al., 2018; Lierl and Hornung, 2003), this may indicate that such interactions primarily influence severe asthma symptoms requiring hospitalization. However, it is possible that outcome misclassification is lower for severe symptoms compared to more moderate symptoms, the latter of which are usually captured using self-reported questionnaires which are subject to recall bias, or surrogate measures such as medication use. This is supported by the results of a panel study on children with asthma which reported a pollen-ozone interaction for daily incident asthma attacks but not for the less severe outcomes considered (daily records of respiratory symptoms, medication use or measures of peak expiratory flow rates) (Just et al., 2002). In this review, the proportion of studies reporting significant allergen-pollutant interactions was higher among those using medical records (9/25) than those using self-reported health outcomes (3/10). However, it should be noted that the studies relying on self-reported health outcomes were all panel or cohort studies with smaller sample sizes and shorter follow-up times than the studies using medical records (i.e. timeseries and case-crossover studies). Disease misclassification is nonetheless possible for hospital administrative data but is unlikely to explain the interactions observed as coding errors or preferences (including missingness) are likely independent of daily changes in allergen or pollution concentrations.

Four interactions were observed for allergic outcomes (three for medical consultations for pollinosis (i.e. allergic rhinitis due to pollen) and one for any allergic (nasal, ocular or bronchial) symptom), which supports a role of allergen-pollutant interactions on allergic pathways. Indeed, even for the outcomes not exclusively related to allergy (e.g. asthma), one must assume that the observed increases in these outcomes on days with high allergen counts are driven by persons who are sensitized to the allergen in question. Among the 12 studies that reported allergen-pollutant interactions, the atopic status of participants is unknown in the six timeseries and case-crossover studies on asthma (Anderson et al., 1998; Cakmak et al., 2012; Dales et al., 2004; Gleason et al., 2014; Guilbert et al., 2018; Lierl and Hornung, 2003) and assumed to be 100% in the three timeseries and case-crossover studies on medical consultations for pollinosis (Konishi et al., 2014; Phosri et al., 2017; Sakata et al., 2017). For the remaining three panel studies, stratification by atopic status was not possible in one as 90.2% of participants were atopic (Just et al., 2002) and the other two with more balanced distributions (63% and 57.2% atopic in (Higgins et al., 2000) and (Kanatani et al., 2016), respectively) did not assess whether allergen-pollutant interactions may be stronger among atopic individuals.

Overall, the epidemiological evidence for allergen-pollution interactions found in this systematic review was relatively weak and inconsistent. Only the interactions between Japanese pollen counts (comprising Japanese cedar and Japanese cypress pollen) and measures of high desert dust levels or particulate matter were reported by more than one study (Kanatani et al., 2016; Konishi et al., 2014; Phosri et al., 2017; Sakata et al., 2017). This contrasts with the rather strong and consistent evidence from large epidemiological studies suggesting independent effects of air pollution (Orellano et al., 2017) and allergens (Erbas et al., 2018) on respiratory health, as well as that from small experimental studies in humans demonstrating that allergen and air pollution (particularly diesel exhaust particles) co-exposures alter immune responses as well as gene and microRNA expression, induce epigenetic changes, increase inflammation and reduce lung function in allergic and asthmatic individuals (Barck et al., 2002; Carlsten et al., 2016; Clifford et al., 2017; Hosseini et al., 2016; Mookherjee et al., 2018; Rider et al., 2016; Svartengren et al., 2000; Wooding et al., 2019).

There are several reasons that may explain the predominantly null and inconsistent epidemiological findings observed. Inadequate statistical power to detect interactions is suspected in some studies, as larger samples sizes are typically required to observe a statistical interaction than a main effect of equal magnitude (Hunter, 2005; VanderWeele and Knol, 2014). Although most of the timeseries and case-crossover studies that reported significant allergen-pollutant interactions had large sample sizes and long follow-ups, this was also the case for several of the studies that did not report significant interactions. As small sample sizes, few daily cases or a short follow-up period all contribute to low statistical power, these may be reasons why interactions were more commonly detected in the timeseries or case-crossover studies than in the panel studies, the latter of which are more likely to suffer from these issues. However, well-designed panel studies conducted in high risk (i.e. atopic) individuals could nonetheless be sufficiently powered if conducted in areas and during months with high levels and variation in allergen and pollutant concentrations over short periods of time. Indeed, several authors highlighted low levels or small variation in exposures as limitations of their analyses (Babin et al., 2008; Babin et al., 2007; Darrow et al., 2012; Delfino et al., 2002; Delfino et al., 1997; Jalaludin et al., 2000; Krmpotic et al., 2011; Lewis et al., 2000; Lierl and Hornung, 2003; Villeneuve et al., 2006). Peak allergen and air pollution times may also not coincide for certain combinations, which may explain why interactions are observed for some allergens but not others (Cakmak et al., 2012).

For most studies reviewed, daily allergen and pollution exposures were assessed using data from one or a few central monitoring sites and a single value assigned to the whole study area, which is unlikely to capture true variation in individual-level exposures. Error in attributing exposure based on this method would likely be random, however it is difficult to confirm how this would affect the allergen-pollutant interactions tested (e.g. reductions in available statistical power). The size of this measurement error will vary by study area due to heterogeneity of exposure sources, weather conditions and physical barriers that affect transport of allergens or pollutants, and is expected to be larger for primary pollutants that vary more over smaller spatial scales (e.g. those from traffic emissions) than secondary pollutants (e.g. ozone). Most studies used volumetric traps (Burkard/Hirst) to collect allergen data while some used Rotorod, Durham or Cour samplers, in addition to other methods. These samplers have been shown to be comparable, although the Rotorod is less efficient at sampling small particles (<10 μm in diameter) (Frenz, 1999). Hence, fungal spore counts in studies which used Rotorod samplers may be underestimated (Jones et al., 1994; Lierl and Hornung, 2003; Ross et al., 2002).

Further sources of exposure misclassification relate to individual-behaviour (e.g. time-activity patterns and time spent indoors/outdoors). The potential confounding effect of other outdoor allergens (i.e. fungal allergens on pollen allergen associations and vice versa) as well as indoor allergens was rarely considered, which may not be an issue in all study areas as various allergen species have peak concentrations at different times of the year.

It is worth noting that all studies included in this systematic review (and that recently conducted on the main effects of outdoor pollen on asthma emergency department presentations (Erbas et al., 2018)) used daily pollen grain or fungal spore *counts* as the measure of allergen exposure, rather than measured airborne allergen *levels* (amount of airborne bioallergenic material). Each pollen grain can release a different amount of pollen allergen into the air (up to a ten-fold daily variation) related to an interplay of external and meterological variables and in response to various environmental factors (Buters et al., 2015; Plaza et al., 2016). Similar observations have been made for fungal spores (e.g. *Alternaria* (Grewling et al., 2019)). The role of air pollutants on this day-by-day variation in allergen levels remains poorly understood. Allergen levels are also likely to differ by species/cultivars (Jung et al., 2018). The lack of precise information on allergen levels in the air may have contributed to the null findings. It is also possible that peaks in allergen levels (or allergen counts) are more important than daily averages and future studies may consider more temporally resolved data to account for hourly exposures.

The search strategy used in this systematic review allowed a broad review of studies investigating all potential allergen-pollutant interactions on respiratory health. It was not designed to capture all studies examining independent effects of air pollutants or allergens on respiratory health. One difficulty of conducting systematic reviews on interaction effects is that testing for interactions is frequently done as a secondary analysis (17/35 of the included studies mentioned “interactions” in their study aims). Hence, we may have missed studies that tested for allergen-pollutant interactions but did not report the results (during our search we identified at least three such studies (Caillaud et al., 2014a; Caillaud et al., 2014b; Delfino et al., 1998)). Furthermore, as we restricted our search to studies that evaluated interactions with measured outdoor allergen counts, we excluded those that assessed interactions with “season” only, as a “season” term could be a marker of several environmental factors, and those that used various methods to define pollen seasons (e.g. Google Trends (Bédard et al., 2020) or predictive modelling (Di Menno di Bucchianico et al., 2019)).

Although a meta-analysis was originally invisioned, this was not possible as so few studies reported effect estimates for all interaction terms tested. Several studies reported interaction terms as simply “non-significant”. Of the studies that did report numerical results, very few overlapped in terms of allergen and pollutant considered and all reported at least some significant interactions, suggesting that any combined meta-analytic result would be subject to reporting bias and consequently be biased towards a significant finding. Even comparing available studies at a descriptive level is challenging due to differences in the study period and population, outcomes and exposures considered, and assessment methods used, as well as the statistical strategy pursued. Differences in the prevalence of atopy, cultural habits regarding medication and hospital services use, heterogeneity in allergen species and allergenicity as well as air pollution concentrations and sources further complicate any geographical comparisons.

## 5 Conclusions

In summary, the epidemiological evidence supporting interactions between allergens and air pollutants in the context of respiratory health at the population-level is relatively weak. A third of studies reviewed reported statistically significant interactions, nearly half of which were from timeseries/case-crossover studies on daily number of asthma hospital admissions or visits. Future studies investigating allergen-pollutant interactions should ensure sufficient statistical power, conduct subgroup analysis by atopic status, be designed to capture peaks in both allergen counts and pollutant concentrations and investigate variations in the biological allergenic potential (i.e. allergen levels) of the air. As climate change will influence the amount, allergenicity and geographical spread of allergens and increase the concentrations of some pollutants, any adverse effects of air pollutants on the respiratory response to allergens will be relevant for the development of environmental protection policies.

## Funding source

EF is a recipient of the Imperial College Research Fellowship (2019–2023). HL is a recipient of the MRC Centre for Environment and Health Early Career Fellowship (MR/T502613/1).

## Role of funding source

The funding sources had no role in the study design, in the collection, analysis and interpretation of data, the writing of the report and in the decision to submit the article for publication.

## Declaration of competing interest

The authors declare that they have no known competing financial interests or personal relationships that could have appeared to influence the work reported in this paper.

## Appendix A Supplementary data

Supplementary material Image 1
