# Supplementary material for: Interactive effects of allergens and air pollution on respiratory health: A systematic review
Source: Sci Total Environ. 2021 Feb 25;757:143924. doi: 10.1016/j.scitotenv.2020.143924 (PMC7812370; doi:10.1016/j.scitotenv.2020.143924)
Supplement: Supplementary file 1 — Supplementary material [file mmc1.docx]

**SUPPORTING INFORMATION**

**Interactive effects of allergens and air pollution on respiratory health: A systematic review**

Holly CY Lam^1,2^, Debbie Jarvis^1,2^, Elaine Fuertes^1^,

1 - National Heart and Lung Institute, Imperial College London, London, United Kingdom

2 - MRC Centre for Environment & Health, Imperial College, London, United Kingdom

**Search strategy for additional databases**

**EMBASE**

Subject headings have been slightly adapted as “best fit” for EMBASE, by comparing those used in MeSH for Medline

**Table S1:** Search terms used to identify articles in EMBASE

| **Category** | **Search terms** |
| --- | --- |
| Respiratory health | Subject headings: Asthma OR Bronchitis OR Coughing OR Forced Expiratory Volume OR Lung Ventilation OR Abnormal Respiratory Sound OR Rhinitis OR Respiratory Tract Disease OR Spirometry OR Vital CapacityFree text: Airway* OR asthma* OR breath* OR bronchi* OR cough* OR exacerbation* OR hayfever OR hay fever OR lung OR pulmonary OR respiratory OR rhinoc* OR wheez* |
| Outdoor allergens | Subject headings: Allergen OR Alternaria OR Cladosporium OR Fungus OR Pollen  Free text: aeroallergen* OR aero allergen or aero allergens OR allergen* OR aspergillus OR cedar OR grass OR birch OR pollen* OR mould* OR mold* OR parietaria OR ragweed OR spore* |
| Air pollution | Subject headings: Air Pollution OR Air Pollutant OR Ozone OR Nitrogen Dioxide OR Particulate Matter OR Sulfur Dioxide OR [Traffic Pollution](https://www.ncbi.nlm.nih.gov/mesh/2027874) OR [Exhaust](https://www.ncbi.nlm.nih.gov/mesh/68001335) Gas  Free text: air quality OR carbon monoxide OR emission* OR exhaust OR freeway* OR highway* OR motorway* OR nitrogen dioxide OR particulat* OR particle* OR pollut* OR road* OR sulphur dioxide OR sulfur dioxide OR traffic OR ozone |
| Study design | Subject headings: Case Control Study OR Crossover Procedure OR Cohort Analysis OR [Longitudinal Study](https://www.ncbi.nlm.nih.gov/mesh/68008137)  Free text: case control OR casecontrol OR case crossover OR cohort OR daily OR longitudinal OR panel OR timeseries OR time series |

# **Scopus**

As no subject headings are used in SCOPUS, all relevant subject headings changed to keywords

**Table S2:** Search terms used to identify articles in SCOPUS

| **Category** | **Search terms** |
| --- | --- |
| Respiratory health | airway* OR asthma* OR breath* OR bronchi* OR cough* OR exacerbation* OR hayfever OR “hay fever” OR lung OR pulmonary OR respiratory OR rhinitis OR rhinoc* OR spirometry OR wheez* |
| Outdoor allergens | aeroallergen* OR “aero allergen” or “aero allergens” OR allergen* OR alternaria OR aspergillus OR cedar OR cladosporium OR fung* OR grass OR birch OR pollen* OR mould* OR mold* OR parietaria OR ragweed OR spore* |
| Air pollution | “air quality” OR “carbon monoxide” OR emission* OR exhaust OR freeway* OR highway* OR ozone OR motorway* OR “nitrogen dioxide” OR particulat* OR particle* OR pollut* OR road* OR “sulphur dioxide” OR “sulfur dioxide” OR traffic OR ozone |
| Study design | “case-control” OR “case control” OR casecontrol OR “cross-over” OR “case crossover” OR cohort OR daily OR longitudinal OR panel OR timeseries OR “time series” |

**Table S3:** Additional characteristics of studies ordered by study design (timeseries and case-crossover studies, panel studies, cohort study) and year

| **Author, year** | **Type of study** | **Age group** | **Sample size** | **Outcome summary** | **Allergen measurement method** | **Air pollutant measurement method** | **Covariates**^†^ | **Main effects of allergen and air pollutants** |
| --- | --- | --- | --- | --- | --- | --- | --- | --- |
| Guilbert et al. 2018 (1) | Timeseries | All ages; also stratified into: 0-14, 15-59, >60 years | 1.0 to 1.1 million; 5094 asthma hospitalizations | Overall mean daily asthma admissions: 2.3 | One monitoring site in center of study area (Hirst-type volumetric trap) | Daily estimate informed by monitoring sites (N=10) and land-use regression model. One population-weighted daily estimate used for whole study area. | Seasonal and long-term trends, day of the week, public holidays, mean temperature and relative humidity. Influenza and respiratory infections tested in sensitivity analyses. | Asthma admissions associated with grass, birch and hornbeam pollen counts. Effects stronger for those < 60 years for several pollen taxa. |
| Phosri et al. 2017 (2) | Case-crossover | Age not specified; assumed whole population. | 5.1 million residents (health data from 10 clinics); 73,995 visits | Overall mean daily pollinosis clinic visits: 96 | One monitoring site (Durham method) | PM_2.5_ mass from mean of central monitoring sites^‡^. PM_2.5_ component data from one monitoring site on Fukuoka University roof. | Mean temperature and relative humidity (day of the week, long-term trends and season effects controlled by design). | Visits for pollinosis associated with pollen counts. |
| Sakata et al. 2017 (3) | Timeseries | Age not specified; assumed whole population. | Size of at-risk population not reported; 65,488 visits; asian dust days on 238 of 3,630 days | Overall mean daily pollinosis clinic visits: 9.2 | One monitoring site (Durham method) | Asian dust days (yes/no) provided by Japan Meteorological Agency. Mean of 4 monitoring sites for suspended particulate matter. | Day of the week, public holidays, mean temperature and relative humidity, month and year. | Visits for pollinosis associated with presence of asian dust days and (more weakly) suspended particulate matter. |
| Tham et al. 2017a (4) | Case-crossover | 2-18 years (mean age 5.5 years) | Size of at-risk population not reported; 2098 children included | Total of 1180, 885 and 33 hospitalizations in the three hospitals, respectively | One monitoring site on one of the included hospitals (Burkard trap) | One monitoring site in one of the cities, 20km from allergen measurement site | Maximum temperature and mean relative humidity | In overall population, asthma hospitalizations weakly associated for certain lags of *Coprinus*, *Periconia*, and *Chaetomium* and total fungal spores counts. Some associations stronger among females and older adolescents (14-18 years). |
| Tham et al. 2017b (5) | Case-crossover | 2-17 years (median age 5.2 years); also stratified into: 1) 2-5, 6-10, 11-15, 16-18 years and 2) 2-14, 15-18 years | Size of at-risk population not reported; 644 children included | 644 children with asthma | One monitoring site 2km away from recruiting hospital (Burkard trap) | Several monitoring stations in study area^‡^ | Maximum temperature and mean relative humidity, human rhinovirus infection. Sensitization status also tested but not found to be relevant covariate. | In overall population, asthma hospitalizations associated with same-day *Alternaria, Leptosphaeria, Coprinus, Drechslera*, and total spore counts. Some associations stronger for those with *Cladosporium* sensitization. Additional associations with other fungal taxa seen with lags up to 3 days. |
| Chen et al. 2016 (6) | Timeseries and case-crossover | All ages; also stratified into: < 17 vs > 18 years | Size of at-risk population not reported; 3653 observation-days, of which 388 had missing exposure data. | Overall mean daily asthma hospital admissions: 9.9 | One monitoring site (volumetric Hirst trap) | One monitoring site 5km west of city center | For timeseries: day of the week, weekend/public holidays vs not, mean temperature and humidity, long-term time trends, seasonality. For case-crossover: mean temperature and humidity | Asthma admissions associated with NO_2_, PM_2.5_, PM_10_ and pollen counts, with effects stronger in cold season. O_3_ effects stronger in warm season. Effect estimates strongest in children. |
| Gleason et al. 2014 (7) | Case-crossover | 3-17 years | Size of at-risk population not reported; 21,854 asthma visits | Overall total asthma visits: 21,854 | One monitoring site (in Springfield, volumetric trap) | Spatial and temporal model (12km x 12km resolution) | 3-day average temperature and relative humidity, holiday indicator, school-in-session indicator (as marker of influenza) | Asthma visits associated with O_3_, PM_2.5_, and tree and weed pollen counts. Only minimal associations with grass pollen counts. |
| Konishi et al. 2014 (8) | Case-crossover | Age not specified; assumed whole population. | Size of at-risk population not reported; 11,713 consultations | Overall mean daily consultations: 10.6 | 9 monitoring sites (Durham method) | Several monitoring sites^‡^ | Mean temperature and relative humidity (day of the week, long-term and seasonal effects adjusted by design, public holidays and weekends excluded) | Main effects of pollen counts or pollutants not reported. |
| Cakmak et al. 2012 (9) | Timeseries | All ages. | Over 12 million people. | Overall mean daily asthma hospital admissions: 28.7 | One monitoring site per city (rotational impaction). Same method used in each city. | Monitoring sites in each city^‡^ | Day of the week, mean temperature, barometric pressure and relative humidity. | Asthma admissions reported to be associated with all allergen counts (results not presented). |
| Darrow et al. 2012 (10) | Case-crossover | All ages; also stratified into: 1-4, 5-17, >18 years | Size of at-risk population not reported; 1408 to 1782 observation-days depending on pollen species. | Overall total visits for asthma and wheeze: 400,819 | One monitoring site on rooftop (Rotorod sampler). Data measured five days/week, and estimated for Friday and Saturday | Monitoring sites^‡^ | Month, year, maximum and minimum temperature, average dew point, hospital, day of week, holidays, upper respiratory infections | Associations with asthma- and wheeze-related visits strongest for Quercus and grass pollen counts. |
| Erbas et al. 2012 (11) | Timeseries | < 15 years | Size of at-risk population not provided; 2559 asthma visits recorded | Overall mean daily asthma visits: 21 | One monitoring site on University of Melbourne | One monitoring site (~7km NE of Melbourne CBD) | Maximum temperature, mean relative humidity, rainfall, time trend, day of the week | Asthma visits associated with grass pollen counts, even at low levels. |
| Ghosh et al. 2012 (12) | Timeseries | Age not specified; assumed whole population. | 16.6. million people in Kolkata (health data from two state-run teaching hospitals included); 180 10-day observation-slots available | Mean daily asthma-related admissions were highest in March (21.8) and September (22.3) and lowest in January (2.8) and July (2.9) | One monitoring site (Burkard trap) located 16-20 miles from the two hospitals | One monitoring site | None mentioned | Asthma admissions associated with *Cheno-Amaranthaceae* and *Cyperaceae* pollen counts, SO_2_ and respirable particulate matter. |
| Krmpotic et al. 2011 (13) | Timeseries | >18 years | > 779,000; 808 asthma admissions recorded | Overall mean daily asthma visits: 0.7 | Measurement method not stated. Data from the Institute of Public Health. | Data from 4 monitoring stations^‡^ | Temperature, humidity, day of the week, season, weekly influenza cases | Asthma admissions associated with CO, NO_2_ and hornbeam pollen counts. |
| Babin et al. 2008 (14) | Timeseries | All ages; also stratified into: 5-12, 21-49 years | Size of at-risk population not reported; 61,218 visits recorded | Overall total asthma-related visits: 61,218 | Measurement method not stated. Data from the US Army Centralized Allegen Extract Laboratory. Pollen collected > 3 days/week. | Monitoring sites in DC, ~3 per pollutant | Long-term trend, day of the week, tempeature and dew point | Overall asthma visits associated with PM_2.5_. In warm season, visits associated with O_3_, grass and tree pollen (all ages), O_3_ and grass (5-12 year-olds), and O_3_ (21-49 year-olds). |
| Babin et al. 2007 (15) | Timeseries | 0-17 years; also stratified into: 1-4; 5-12; 12-17 years | Size of at-risk population not reported. | Annual rate of asthma emergency department visits for 1-17 year-olds: 0.029 | Measurement method not stated. Data from the US Army Centralized Allegen Extract Laboratory. Pollen collected ~ 3 days/week (more frequently in Spring/Fall) | Monitoring sites in DC, 3 per pollutant | Maximum temperature, month and season (day of week not found to be relevant) | Asthma visits associated with tree pollen counts for 5-12 year-olds, weed pollen counts for 5-12 and 1-17 year-olds, and O_3_ for 1-17 year-olds. Latter associations strong for 5-12 year-olds. |
| Carracedo‐Martinez et al. 2008 (16) | Case-crossover | All ages | Approximately 300,000 | Overall mean daily calls for cardiovascular and respiratory causes were ~4 and 3. | One monitoring site on top of city hall (volumetric trap) | Average of 8 monitoring sites | Mean temperature, atmospheric pressure and relative humidity, Sunday/public holiday vs not, weekly cases of influenza | Calls for respiratory causes associated with several types of pollen counts. Calls for cardiovascular, respiratory and cardiorespiratory causes associated with black smoke and SO_2_. |
| Villeneuve et al. 2006 (17) | Timeseries | > 65 years | Size of at-risk population not reported. | Total primary care visits for allergic rhinitis: 52,691 | One monitoring site (rotational impaction) | Monitoring sites (ranging from 4-10 sites depending on pollutant) | Long-term time trends (adjusted for day of the week and public holidays), influenza visits, mean daily temperature and relative humidity | Allergic rhinitis visits associated with 10-day average ragweed counts but none of the pollutants tested. |
| Dales et al. 2004 (18) | Timeseries | All ages; also stratified into: < 13 vs >13 years (median) | 7.83 million;  60,066 asthma admissions recorded | Total asthma hospital admissions: 60,066 | One monitoring site per city. Same method used in each city. | Monitoring sites in each city (rotational impaction)^‡^ | Day of the week, long-term time trends, weather variables (maximum and minimum temperatures, mean barometric pressure, dew point temperature, and relative humidity) | In combined analyses, asthma admissions associated with weed and grass pollen counts and all three fungal spores counts. City-specific associations consistently positive and majority significant. |
| Lierl and Hornung 2003 (19) | Timeseries | Children; age not specified | Size of at-risk population not reported. | Monthly daily means ranged from 5.5 to 17.2 | One monitoring site (Rotorod sampler) | One monitoring site | None listed | Asthma visits associated with total pollen counts. |
| Sunyer et al. 2002 (20) | Case-crossover | >14 years | Size of at-risk population not reported; 1078 participants died | 1078 participants died of the 5610 captured in study sample. Main causes of death were cardiovascular in women and respiratory in men admitted once, and respiratory in both sexes among those admitted more than once. | One monitoring site in NE of city (Cour method) | Mean of 3 monitoring sites, except black smoke which was assessed using network of 15 samplers. | Temperature, humidity, hot days, influenza epidemics and soybean asthma epidemics. Day of the week controlled for by design. | Among severe asthmatics, risk of mortality associated with NO_2_. Similar associations observed with O_3_ but only in warm season. |
| Weisel et al. 2002 (21) | Timeseries | Age not specified; assumed whole population. | Size of at-risk population not reported. | Summary of outcomes not provided | Measurement method not stated. Data obtained from the University Hospital, Newark. | Mean of several monitoring stations in study area^‡^ | Temperature, relative humidity | Asthma visits and admissions associated with pollen counts and O_3_. |
| Lewis et al. 2000 (22) | Timeseries | >14 years | 400,000 | Overall mean daily asthma admissions and visits were 0.81 and 1.24, respectively | One monitoring site (Burkard trap) 4 km SW of city centre | One monitoring site for O_3_ and NO_2_, and another for black smoke (1 mile away) | Two time trend variables, temperature, humidity, thunderstoms, influenza epidemics and day of the week | Asthma admissions associated with *Cladosporium* counts (weaker associations also seen with asthma visits). Asthma visits associated with grass pollen counts, especially on days with light rainfall. |
| Anderson et al. 1998 (23) | Timeseries | All ages; also stratified into: 0-14, 15-64, > 65 years | Size of at-risk population not reported; 1796 observation-days but pollen only measured on 915 days in warm season. | Overall mean daily asthma admissions: 35.1 | One monitoring site in North London (Burkard trap) | Monitoring sites in London (ranging from 1-4 sites depending on pollutant) | Time trend, seasonal cycles, day of the week, public holidays, influenza epidemics, temperature and humidity | Asthma admissions associated with O_3_ for 15-64 year-olds, NO_2_ for 0-14 and 65+ year-olds, SO_2_ for 0-14 year-olds and black smoke for > 65 year-olds. |
| DellaValle et al. 2012 (24) | Panel | 4-12 years | 430 participants | Median percent of days with any symptom during pollen season: 4.9 (medication users) and 0.90 (medication non-users). | Temporal and spatial model generating daily estimates for each subject based on home residence. | Monitoring sites (range of 8 to 18 depending on pollutant). Values from closest monitor assigned to each participant (living within ~10-21 kms depending on pollutant) | Maximum temperature, season and antibiotic use. | Several asthma symptoms associated with weed pollen count among those sensitized and taking medication. Several asthma symptoms associated with grass pollen counts among those sensitized and not taking medication. |
| Chen et al. 2011 (25) | Panel | Mean age: 10.6 years | 33 asthmatics, 30 with allergic rhinitis, and 37 healthy controls ; 682 observation- days | Average FVC, FEV_1_, FEF_25_, FEF_50_, FEF_75_, FEF_2575_ were 1.98 l, 1.79 l, 3.70 l/s, 2.67 l/s, 1.38 l/s and 2.4 l/s | One monitoring site (Burkard 7-day recording trap). | One monitoring site within 2.5km of schools | Age, height, respiratory infection, asthma/allergic rhinitis symptoms, medicinal use, temperature, relative humidity, day of week, gender, school, parental education, parental atopy, secondhand smoke | Lower FVC and FEV_1_ associated with total fungal spore counts. Lower FVC associated with PM_2.5_ and lower FEF_25_, FEF_50_, FEF_75_, FEF_2575_ associated with O_3_. |
| Jalaludin et al. 2004 (26) | Panel | Mean age: 9.6 years | 125 children; 36,956 observation days | Overall prevalences were 9.7% 10.1, 11.2 for wheeze, wet cough, and dry cough. 14.2% and 16.3% of days had reported use inhaled b2-angonist and inhaled corticosteroids, and 0.5% of days had doctor visits for asthma | Average of 2 monitoring sites used (Burkard spore trap) | Children assigned value from closest of 6 monitoring sites to their school | Time trend, mean temperature and humidity, number of hours spent outdoors, season. | Wet cough associated with NO_2._ Doctor visits for asthma associated with PM_10_. |
| Delfino et al. 2002 (27) | Panel | 9-19 years | 22 participants; 1248 observation-days | Average symptom score was 0.9 and 1.24 (range is 0-6) for those taking and not taking anti-inflammatory medication, respectively. | One monitoring site (Burkard trap) | One monitoring site | Temperature, relative humidity, day of the week, linear time trend, lower and upper respiratory infections | Asthma symptoms associated with air pollutants, pollen and fungal spore counts, although restricted to small group (N=12) not taking anti-inflammatory medications. |
| Just et al. 2002 (28) | Panel | Mean age: 10.9 years | 82 children | Frequency of incident and prevalent episodes of asthmatic attacks were 1.0 and 1.9, respectively. | Measurement method not stated. Data obtained from French surveillance system. | Average of monitoring sites (5 to 22 sites depending on pollutant). | Time trends, day of the week, pollen temperature and relative humidity. Respiratory infections tested in sensitivity analysis. | In overall population, black smoke and NO_2_ associated with nocturnal cough and respiratory infections, O_3_ with asthma attacks, respiratory infections and eye irritation and changes in PEFR, and PM_13_ with eye irritation. |
| Ross et al. 2002 (29) | Panel | 5-49 years | 40 participants; follow-up period is 155 days | Summary of outcomes not provided | 3 monitoring sites placed in study area (Rotorod sampler). Unknown how data combined across sites. | Several monitoring stations in study area.^‡^ Participants lived within 8 km of monitors. | Maximum temperature (relative humidity also adjusted for in models for evening PEFR only) | Total pollen counts and O_3_ associated with increased morning and evening symptom scores, decreased evening PEFR and increased asthma medication use. *Curvularia* counts associated with increased morning and evening symptom scores, decreased morning PEFR and increased asthma medication use. Ragweed pollen counts associated with decreased evening PEFR and increased medication use. Grass pollen counts associated with increased morning and evening symptom scores. Atopic subgroup not found to be at increased risk. |
| Higgins et al. 2000 (30) | Panel | 20-71 years (mean age: 46 years) | 35 patients with asthma or chronic obstructive pulmonary disease | Mean and range of baseline FEV_1_: 2.44 (1.40 – 4.05) | One monitoring site (Burkard spore trap) | Two monitoring sites in each of the two towns. Unclear how exposures assigned to participants. | None listed | O_3_ assocaited with decreasing mean PEFR, increasing variability in PEFR and wheeze symptoms. Fungal spore counts associated with increasing variability in PEFR and wheeze symptoms. NO_2_ associated with increasing wheeze symptoms. Atopic subgroup not at increased risk. |
| Jalaludin et al. 2000 (31) | Panel | Mean age: 9.6 years | 125 children; 31,209 observation-days | Overall mean PEFR was 323.5 L/min (for individual children ranged from 212 to 446) | Average of 2 monitoring sites used (Burkard spore trap) | Average of 6 monitoring sites (population regression model) and value from closest of 6 monitoring sites to school (GEE model) | Time trend, mean temperature and humidity, number of hours spent outdoors, season. | Daily mean deviation in PEFR negatively associated with mean daytime O_3_. Effects stronger in those with bronchial hyperreactivity and asthma. |
| Delfino et al. 1997 (32) | Panel | 9-46 years | 22 participants (9 adults and 13 children); 1218 observation-days | Summary data for overall participants not reported | One monitoring site (Burkard trap) | Personal O_3_ measured using passive sampler. Outdoor O_3_ and other pollutants measured with one monitoring site | Maximum temperature, mean relative humidity, day of the week, respiratory infections. Age, gender, and height controlled for by design. | All 3 outcomes associated with total fungal spores counts. Associations larger for counts of certain fungal types and among those sensitized to the types of fungi measured. Inhaler use associated with PM_10_. |
| Delfino et al. 1996 (33) | Panel | 9-16 years | 12 participants; 462 observation-days  (only 7 participants and 276 observation-days available for inhaler use analyses) | Summary data for overall participants not reported | One monitoring site (Burkard trap) | Personal O_3_ measured using passive sampler. Outdoor O_3_ and other pollutants measured with one monitoring site | Maximum temperature, mean relative humidity, day of the week, respiratory infections. Age, gender, and height controlled for by design. | Symptom scores and inhaler use associated with personal measurements of O_3_ and total fungal spore counts. Inhaler use also associated with outdoor 12-hr O_3_. No clear pattern for those sensitized to fungi. |
| Jones et al. 1994 (34) | Panel | Mean age: 10.6 years | 20 children; follow-up period is 109 days | Daily mean values were 324.8 L for PEFR, 0.4 symptoms per day, 0.5 on symptom scale (range 0-6) and 2 medications used | One monitoring site (Rotorod sampler) | Average of 3 monitoring sites | Maximum temperature, humidity | PEFR, activity time and outside time negatively associated with mold. |
| Kanatani et al. 2016 (35) | Longitudinal cohort | Mean age: 32.2, 31.0, and 30.9 years in Kyoto, Toyama and Tottori, respectively | 3328 pregnant women | Symptoms reported in 48.2% of responses (56.1% and 46.9% of responses on dust and control days, respectively) | Daily mean of closest monitoring station from home (5 stations using beta-ray attenuation method and 3 stations using Durham method). | Measured using a light detection and ranging system (LIDAR). | Smoking status of participants and their partners, house income, age, body mass index, air pressure, temperature and humidity | Risk of allergen symptoms higher on desert-dust days. Effects greater among those sensitized to Japanese cedar pollen when pollen was present in the air. Allergen symptoms associated with high pollen count days. |

^†^other than terms included for allergens and air pollutants; , ^‡^unclear how many monitors or how data were combined/used

PEFR: peak expiratory flow rate

**Table S4:** Summary of quality assessment, ordered by study design (timeseries and case-crossover studies, panel studies, cohort study) and year

|  |  | **Sampling** | | | **Valid measurement** | | | **Data analysis** | | | **Result interpretation** | |  |
| --- | --- | --- | --- | --- | --- | --- | --- | --- | --- | --- | --- | --- | --- |
| **Author, year** | **Study population well described** | **Sampling frame described** | **Eligibility criteria listed** | **Entire eligible population included** | **Allergens** | **Air pollutants** | **Outcomes** | **Data analysis clear** | **Interactions tested explicitly listed**^†^ | **Control for study design** | **> 80% of completed study**^‡^ | **Good confounder control** | **Comments** |
| Guilbert et al. 2018 (1) | Yes | Yes | Yes | Yes | Yes | Yes | Yes | Yes | Yes | Yes | NA | Yes |  |
| Phosri et al. 2017 (2) | No | Yes | Yes | Yes | Yes | Yes | Yes | Yes | Yes | Yes | NA | Yes | No information on ages included. |
| Sakata et al. 2017 (3) | No | Yes | Yes | Yes | Yes | Yes | Yes | Yes | Yes | Yes | NA | Yes | No information on ages included. |
| Tham et al. 2017a (4) | Yes | Yes | Yes | Yes | Yes | Yes | Yes | Yes | Yes | Yes | NA | Yes |  |
| Tham et al. 2017b (5) | Yes | Yes | Yes | Yes | Yes | Yes | Yes | Yes | Yes | Yes | NA | Yes |  |
| Chen et al. 2016 (6) | Yes | Yes | Yes | Yes | Yes | Yes | Yes | Yes | No | Yes | NA | Yes | Text implies allergen-pollutant interactions tested but unclear which combinations examined. |
| Gleason et al. 2014 (7) | Yes | Yes | Yes | Yes | Yes | Yes | Yes | Yes | Yes | Yes | NA | Yes |  |
| Konishi et al. 2014 (8) | No | Yes | Yes | Yes | Yes | Yes | Yes | Yes | Yes | Yes | NA | Yes | No information on ages included. |
| Cakmak et al. 2012 (9) | Yes | Yes | Yes | Yes | Yes | Yes | Yes | Yes | Yes | Yes | NA | Yes |  |
| Darrow et al. 2012 (10) | Yes | Yes | Yes | Yes | Yes | Yes | Yes | Yes | Yes | Yes | NA | Yes |  |
| Erbas et al. 2012 (11) | Yes | Yes | Yes | Yes | Yes | Yes | Yes | Yes | Yes | Yes | Yes | Yes |  |
| Ghosh et al. 2012 (12) | No | Yes | Yes | Yes | Yes | Yes | Unsure | No | No | Yes | NA | No | No information on ages included or outcome definition, other than recorded in 10-day timeslots. Unclear how daily allergen and pollutant data combined into 10-day timeslots. Text implies allergen-pollutant interactions tested but unclear which combinations examined. No confounders listed. |
| Krmpotic et al. 2011 (13) | Yes | Yes | Yes | Yes | Unsure | Yes | Yes | Yes | Yes | Yes | NA | Yes | No information regarding pollen collection method, other than provided by “Institute of Public Health”. |
| Babin et al. 2008 (14) | Yes | Yes | Yes | Yes | Unsure | Yes | Yes | Yes | No | Yes | NA | Yes | No information regarding pollen collection method, other than provided by US Army Centralized Allergen Extract Laboratory. Text implies allergen-pollutant interactions tested but unclear which combinations examined. |
| Babin et al. 2007 (15) | Yes | Yes | Yes | Yes | Unsure | Yes | Yes | Yes | Yes | Yes | NA | Yes | No information regarding pollen collection method, other than provided by US Army Centralized Allergen Extract Laboratory. |
| Carracedo‐Martinez et al. 2008 (16) | Yes | Yes | Yes | Yes | Yes | Yes | Yes | Yes | No | Yes | NA | Yes | Text implies allergen-pollutant interactions tested but unclear which combinations examined. |
| Villeneuve et al. 2006 (17) | Yes | Yes | Yes | Yes | Yes | Yes | Yes | Yes | Yes | Yes | NA | Yes |  |
| Dales et al. 2004 (18) | Yes | Yes | Yes | Yes | Yes | Yes | Yes | Yes | No | Yes (city-specific analyses) /  No (overall analyses) | NA | Yes | Text implies allergen-ozone interactions tested but unclear if other pollutants examined. City specific results presented in addition to overall analyses, the latter of which are not adjusted for differences between cities. |
| Lierl and Hornung 2003 (19) | No | Yes | No | Yes | Yes | Yes | Yes | Yes | Yes | Yes | NA | No | Analysis restricted to children but no age-cut-off specified. No covariates other than air pollution mentioned. |
| Sunyer et al. 2002 (20) | Yes | Yes | Yes | Yes | Yes | Yes | Yes | Yes | No | Yes | NA | Yes | Stated that all allergen – pollutant interactions tested but results only mentioned for NO_2_ and O_3_ (and not for SO_2_ and CO), possibly because main effects only found for NO_2_ and O_3_. |
| Weisel et al. 2002 (21) | No | Yes | Yes | Yes | Unsure | Yes | Yes | Yes | No | Yes | NA | No | No information on ages included or pollen collection method, other than provided by the “University Hospital”. Text implies allergen-pollutant interactions tested but unclear which combinations examined. Time trends in data not accounted for. |
| Lewis et al. 2000 (22) | Yes | Yes | Yes | Yes | Yes | Yes | Yes | Yes | Yes | Yes | NA | Yes |  |
| Anderson et al. 1998 (23) | Yes | Yes | Yes | Yes | Yes | Yes | Yes | Yes | Yes | Yes | NA | Yes |  |
| DellaValle et al. 2012 (24) | No | Yes | Yes | Yes | Yes | Yes | Yes | Yes | Yes | Yes | Yes | Yes | Length of follow-up not specified in article (information provided by corresponding author upon request). |
| Chen et al. 2011 (25) | Yes | Yes | No | Yes | Yes | Yes | Yes | Yes | No | Yes | Yes | Yes | Of 3937 who answered study questionnaire, only 100 participated in lung function testing. Random sample taken but unclear who was eligible or why restricted to 100. Text implies allergen-pollutant interactions tested but unclear which combinations examined. |
| Jalaludin et al. 2004 (26) | Yes | Yes | Yes | No | Yes | Yes | Yes | Yes | Yes | Yes | Yes | Yes | 148 recruited but only 125 included due to study withdrawal and non-compliance. |
| Delfino et al. 2002 (27) | Yes | Yes | Yes | No | Yes | Yes | Yes | Yes | Yes | Yes | Yes | Yes | 25 recruited but only 22 included due to study inclusion criteria and study withdrawal. |
| Just et al. 2002 (28) | Yes | Yes | Yes | No | Unsure | Yes | Yes | Yes | Yes | Yes | Yes | Yes | 100 recruited but only 82 included due to study withdrawal and non-compliance. No information regarding pollen collection method, other than provided by two French surveillance systems. |
| Ross et al. 2002 (29) | Yes | Yes | No | No | Yes | Yes | Yes | No | Yes | Yes | No | Yes | Eligibility criteria unclear. 59 recruited but only 40 included due to study withdrawal and non-compliance. Unclear how data from 3 allergen monitoring sites (placed in 3 areas in study area) were used to assign exposures. |
| Higgins et al. 2000 (30) | Yes | Yes | Yes | No | Yes | Yes | Yes | Yes | Yes | Yes | Yes | No | 36 recruited but only 35 included in PEFR analysis (30 in wheeze analysis). No covariates mentioned. |
| Jalaludin et al. 2000 (31) | Yes | Yes | Yes | No | Yes | Yes | Yes | Yes | No | Yes | Yes | Yes | 148 recruited but only 125 included due to study withdrawal and non-compliance. Text implies allergen-pollutant interactions tested but unclear which combinations examined. |
| Delfino et al. 1997 (32) | Yes | Yes | Yes | No | Yes | Yes | Yes | Yes | Yes | Yes | Yes | Yes | 24 recruited but only 22 included due to non-compliance. |
| Delfino et al. 1996 (33) | Yes | No | Yes | No | Yes | Yes | Yes | Yes | Yes | Yes | No | Yes | Unclear how participants recruited. 15 recruited but only 12 included in asthma symptom severity analysis due to non-compliance. Only 7 included for analysis on inhaler use as rest did not use inhaler. |
| Jones et al. 1994 (34) | Yes | Yes | No | No | Yes | Yes | Yes | Yes | Yes | Yes | Yes | Yes | Eligibility criteria unclear. 24 volunteered but only 20 included due to study withdrawal and living outside study area. |
| Kanatani et al. 2016 (35) | Yes | Yes | Yes | No | Yes | Yes | Yes | Yes | Yes | No | Yes | Yes | Of 4178 eligible pregnant women in cohort, 3327 included. Of those excluded, unclear how many did not return questionnaire and how many did not experience an Asian dust season before delivery. Potential clustering by city not considered. |

^†^ Not included in (36) but added by authors due to nature of systematic review

^‡^ Not applicable for timeseries and case-crossover studies.

**References**

1. Guilbert A, Cox B, Bruffaerts N, Hoebeke L, Packeu A, Hendrickx M et al. Relationships between aeroallergen levels and hospital admissions for asthma in the Brussels-Capital Region: a daily time series analysis. *Environmental Health* 2018;**17**:35.

2. Phosri A, Ueda K, Tasmin S, Kishikawa R, Hayashi M, Hara K et al. Interactive effects of specific fine particulate matter compositions and airborne pollen on frequency of clinic visits for pollinosis in Fukuoka, Japan. *Environmental Research* 2017;**156**:411–419.

3. Sakata S, Konishi S, Ng CFS, Kishikawa R, Watanabe C. Association of asian dust with daily medical consultations for pollinosis in Fukuoka City, Japan. *Environ Health Prev Med* 2017;**22**:25.

4. Tham R, Katelaris CH, Vicendese D, Dharmage SC, Lowe AJ, Bowatte G et al. The role of outdoor fungi on asthma hospital admissions in children and adolescents: A 5-year time stratified case-crossover analysis. *Environmental Research* 2017;**154**:42–49.

5. Tham R, Vicendese D, Dharmage SC, Hyndman RJ, Newbigin E, Lewis E et al. Associations between outdoor fungal spores and childhood and adolescent asthma hospitalizations. *Journal of Allergy and Clinical Immunology* 2017;**139**:1140-1147.e4.

6. Chen K, Glonek G, Hansen A, Williams S, Tuke J, Salter A et al. The effects of air pollution on asthma hospital admissions in Adelaide, South Australia, 2003–2013: time-series and case–crossover analyses. *Clinical & Experimental Allergy* 2016;**46**:1416–1430.

7. Gleason JA, Bielory L, Fagliano JA. Associations between ozone, PM2.5, and four pollen types on emergency department pediatric asthma events during the warm season in New Jersey: A case-crossover study. *Environmental Research* 2014;**132**:421–429.

8. Konishi S, Ng CFS, Stickley A, Nishihata S, Shinsugi C, Ueda K et al. Particulate matter modifies the association between airborne pollen and daily medical consultations for pollinosis in Tokyo. *Science of The Total Environment* 2014;**499**:125–132.

9. Cakmak S, Dales RE, Coates F. Does air pollution increase the effect of aeroallergens on hospitalization for asthma? *Journal of Allergy and Clinical Immunology* 2012;**129**:228–231.

10. Darrow LA, Hess J, Rogers CA, Tolbert PE, Klein M, Sarnat SE. Ambient pollen concentrations and emergency department visits for asthma and wheeze. *Journal of Allergy and Clinical Immunology* 2012;**130**:630-638.e4.

11. Erbas B, Akram M, Dharmage SC, Tham R, Dennekamp M, Newbigin E et al. The role of seasonal grass pollen on childhood asthma emergency department presentations. *Clinical & Experimental Allergy* 2012;**42**:799–805.

12. Ghosh D, Chakraborty P, Gupta J, Biswas A, Roy I, Das S et al. Associations between pollen counts, pollutants, and asthma-related hospital admissions in a high-density Indian metropolis. *Journal of Asthma* 2012;**49**:792–799.

13. Krmpotic D, Krmpotic D, Luzar-Stiffler V, Rakusic N, Markovic AS, Hrga I et al. Effects of traffic air pollution and hornbeam pollen on adult asthma hospitalizations in Zagreb. *IAA* 2011;**156**:62–68.

14. Babin S, Burkom H, Holtry R, Tabernero N, Davies-Cole J, Stokes L et al. Medicaid patient asthma-related acute care visits and their associations with ozone and particulates in Washington, DC, from 1994–2005. *International Journal of Environmental Health Research* 2008;**18**:209–221.

15. Babin SM, Burkom HS, Holtry RS, Tabernero NR, Stokes LD, Davies-Cole JO et al. Pediatric patient asthma-related emergency department visits and admissions in Washington, DC, from 2001–2004, and associations with air quality, socio-economic status and age group. *Environ Health* 2007;**6**:9.

16. Carracedo‐Martinez E, Sanchez C, Taracido M, Saez M, Jato V, Figueiras A. Effect of short-term exposure to air pollution and pollen on medical emergency calls: a case-crossover study in Spain. *Allergy* 2008;**63**:347–353.

17. Villeneuve PJ, Doiron M-S, Stieb D, Dales R, Burnett RT, Dugandzic R. Is outdoor air pollution associated with physician visits for allergic rhinitis among the elderly in Toronto, Canada? *Allergy* 2006;**61**:750–758.

18. Dales RE, Cakmak S, Judek S, Dann T, Coates F, Brook JR et al. Influence of outdoor aeroallergens on hospitalization for asthma in Canada. *Journal of Allergy and Clinical Immunology* 2004;**113**:303–306.

19. Lierl MB, Hornung RW. Relationship of outdoor air quality to pediatric asthma exacerbations. *Annals of Allergy, Asthma & Immunology* 2003;**90**:28–33.

20. Sunyer J, Basagaña X, Belmonte J, Antó JM. Effect of nitrogen dioxide and ozone on the risk of dying in patients with severe asthma. *Thorax* 2002;**57**:687–693.

21. Weisel CP, Cody RP, Georgopoulos PG, Purushothaman V, Weiss SH, Bielory L et al. Concepts in developing health-based indicators for ozone. *IAOEH* 2002;**75**:415–422.

22. Lewis SA, Corden JM, Forster GE, Newlands M. Combined effects of aerobiological pollutants, chemical pollutants and meteorological conditions on asthma admissions and A & E attendances in Derbyshire UK, 1993–96. *Clinical & Experimental Allergy* 2000;**30**:1724–1732.

23. Anderson HR, Leon AP de, Bland JM, Bower JS, Emberlin J, Strachan DP. Air pollution, pollens, and daily admissions for asthma in London 1987–92. *Thorax* 1998;**53**:842–848.

24. DellaValle CT, Triche EW, Leaderer BP, Bell ML. Effects of ambient pollen concentrations on frequency and severity of asthma symptoms among asthmatic children. *Epidemiology* 2012;**23**:55–63.

25. Chen B-Y, Chao HJ, Chan C-C, Lee C-T, Wu H-P, Cheng T-J et al. Effects of ambient particulate matter and fungal spores on lung function in schoolchildren. *Pediatrics* 2011;**127**:e690–e698.

26. Jalaludin BB, O’Toole BI, Leeder SR. Acute effects of urban ambient air pollution on respiratory symptoms, asthma medication use, and doctor visits for asthma in a cohort of Australian children. *Environmental Research* 2004;**95**:32–42.

27. Delfino R, Zeiger RS, Seltzer JM, Street DH, McLaren CE. Association of asthma symptoms with peak particulate air pollution and effect modification by anti-inflammatory medication use. *Environmental Health Perspectives* 2002;**110**:A607–A617.

28. Just J, Ségala C, Sahraoui F, Priol G, Grimfeld A, Neukirch F. Short-term health effects of particulate and photochemical air pollution in asthmatic children. *European Respiratory Journal* 2002;**20**:899–906.

29. Ross MA, Persky VW, Scheff PA, Chung J, Curtis L, Ramakrishnan V et al. Effect of ozone and aeroallergens on the respiratory health of asthmatics. *Archives of Environmental Health: An International Journal* 2002;**57**:568–578.

30. Higgins BG, Francis HC, Yates C, Warburton CJ, Fletcher AM, Pickering CA et al. Environmental exposure to air pollution and allergens and peak flow changes. *European Respiratory Journal* 2000;**16**:61–66.

31. Jalaludin BB, Chey T, O’Toole BI, Smith WT, Capon AG, Leeder SR. Acute effects of low levels of ambient ozone on peak expiratory flow rate in a cohort of Australian children. *Int J Epidemiol* 2000;**29**:549–557.

32. Delfino RJ, Zeiger RS, Seltzer JM, Street DH, Matteucci RM, Anderson PR et al. The effect of outdoor fungal spore concentrations on daily asthma severity. *Environmental Health Perspectives* 1997;**105**:622–635.

33. Delfino RJ, Coate BD, Zeiger RS, Seltzer JM, Street DH, Koutrakis P. Daily asthma severity in relation to personal ozone exposure and outdoor fungal spores. *Am J Respir Crit Care Med* 1996;**154**:633–641.

34. Jones G, Brantley P, Hebert R, Kidd J, Shadravan I, McClure J et al. Air quality and respiratory functioning in children with pulmonary disorders. *J La State Med Soc* 1994;**146**:455–461.

35. Kanatani KT, Hamazaki K, Inadera H, Sugimoto N, Shimizu A, Noma H et al. Effect of desert dust exposure on allergic symptoms: A natural experiment in Japan. *Annals of Allergy, Asthma & Immunology* 2016;**116**:425-430.e7.

36. Zaza S, Wright-De Agüero LK, Briss PA, Truman BI, Hopkins DP, Hennessy MH et al. Data collection instrument and procedure for systematic reviews in the guide to community preventive services. *American Journal of Preventive Medicine* 2000;**18**:44–74.
